# Systematic Review of the Use of Dried Blood Spots for Monitoring HIV Viral Load and for Early Infant Diagnosis

**DOI:** 10.1371/journal.pone.0086461

**Published:** 2014-03-06

**Authors:** Pieter W. Smit, Kimberly A. Sollis, Susan Fiscus, Nathan Ford, Marco Vitoria, Shaffiq Essajee, David Barnett, Ben Cheng, Suzanne M. Crowe, Thomas Denny, Alan Landay, Wendy Stevens, Vincent Habiyambere, Joseph H. Perriens, Rosanna W. Peeling

**Affiliations:** 1 Department of Clinical Research, London School of Hygiene and Tropical Medicine, London, United Kingdom; 2 Department of Microbiology and Immunology, University of North Carolina, Chapel Hill, North Carolina, United States of America; 3 Department of HIV/AIDS, World Health Organization, Geneva, Switzerland; 4 HIV, Medicine and Science, Clinton Health Access Initiative, New York, New York, United States of America; 5 Department of Haematology, UK NEQAS for Leucocyte Immunophenotyping, Sheffield, United Kingdom; 6 Department of Technology and Innovation, Pangaea Global AIDS Foundation, San Fransisco, California, United States of America; 7 Centre for Biomedical Research, Burnet Institute, Melbourne, Australia; 8 Department of Medicine, Duke Human Vaccine Institute and Center for HIV/AIDS Vaccine Immunology, Durham, North Carolina, United States of America; 9 Department of Immunology- Microbiology, Rush University Medical Center, Chicago, Illinois, United States of America; 10 Department of Molecular Medicine and Haematology, University of the Witwatersrand, Johannesburg, South Africa; 11 Department of HIV/AIDS, World Health Organization, Geneva, Switzerland; 12 Department of Clinical Research, London School of Hygiene & Tropical Medicine, London, United Kingdom; Institute of Infection and Global Health, United Kingdom

## Abstract

**Background:**

Dried blood spots (DBS) have been used as alternative specimens to plasma to increase access to HIV viral load (VL) monitoring and early infant diagnosis (EID) in remote settings. We systematically reviewed evidence on the performance of DBS compared to plasma for VL monitoring and EID.

**Methods and Findings:**

Thirteen peer reviewed HIV VL publications and five HIV EID papers were included. Depending on the technology and the viral load distribution in the study population, the percentage of DBS samples that are within 0.5 log of VL in plasma ranged from 52–100%. Because the input sample volume is much smaller in a blood spot, there is a risk of false negatives with DBS. Sensitivity of DBS VL was found to be 78–100% compared to plasma at VL below 1000 copies/ml, but this increased to 100% at a threshold of 5000 copies/ml. Unlike a plasma VL test which measures only cell free HIV RNA, a DBS VL also measures proviral DNA as well as cell-associated RNA, potentially leading to false positive results when using DBS. The systematic review showed that specificity was close to 100% at DBS VL above 5000 copies/ml, and this threshold would be the most reliable for predicting true virologic failure using DBS. For early infant diagnosis, DBS has a sensitivity of 100% compared to fresh whole blood or plasma in all studies.

**Conclusions:**

Although limited data are available for EID, DBS offer a highly sensitive and specific sampling strategy to make viral load monitoring and early infant diagnosis more accessible in remote settings. A standardized approach for sampling, storing, and processing DBS samples would be essential to allow successful implementation.

**Trial Registration:**

PROSPERO Registration #: CRD42013003621.

## Introduction

According to the latest WHO figures, 34 million people were living with HIV worldwide in 2011 [1]. As of mid-2012, over eight million HIV infected individuals have been placed on antiretroviral therapy (ART) and will require ongoing monitoring to ensure treatment continues to be efficacious [2]. While CD4 cell count has traditionally been used to monitor patients on treatment in resource-limited settings, HIV viral load (VL) testing is increasingly recognized as the preferred tool for monitoring treatment efficacy, detecting treatment failure, and preventing misclassification of treatment responses and inappropriate switching of treatment regimens [3]. However, despite recommendations by WHO in 2010 for countries to begin phasing in VL monitoring, current VL technology remains out of reach for the majority of patients in most resource-limited settings as standard HIV nucleic acid approaches depend on expensive equipment, dedicated laboratory space and highly trained laboratory technologists [4], [5].

Similarly, access to HIV nucleic acid detection for confirmation of early infant diagnosis of HIV infection is a challenge. Antibody detection assays cannot be used for infants less than 18 months of age as maternal IgG antibodies can cross the placental barrier and yield false positive results. Assays to detect HIV nucleic acids are more technically demanding and costly than simple antibody tests and are neither affordable nor widely available [4], [5]. Among these, the most promising is the use of dried blood spots (DBS). The process of DBS collection begins with a finger or heel prick and spotting whole blood directly onto filter paper, which is then left to dry at room temperature. Once dried, DBS can be stored with desiccant and shipped to central laboratories for HIV virologic testing. DBS has several advantages over traditional methods of sample collection: there is no need for phlebotomy; DBS increases the accessibility of HIV VL and EID testing in remote areas; it is a relatively easy and inexpensive means of collecting and storing samples under field conditions; DBS is easy to transport from peripheral site to central laboratory; and DBS reduces materials required, biological waste produced and sampling costs for specimen collection compared to venipuncture [6]–[10]. The use of DBS as an alternative sampling method for HIV VL and EID assays may influence sensitivity and accuracy [11].

## Methods

We performed a systematic review of studies evaluating and comparing the performance of DBS samples to plasma for HIV VL quantification, and DBS for EID to whole blood (Appendix S1).

### Eligibility criteria

Eligibility criteria were defined using the PICOS format (Population, Interventions, Comparisons, Outcomes, Study Design). Studies evaluating DBS and plasma samples for HIV VL measures with commercially available technologies at the time of the review (April 2012) were considered for inclusion.

### Search strategy

Studies published between January 1998 and April 2012 were identified by a search of MEDLINE and Embase databases. A separate search strategy was used for HIV VL search and EID as defined in the study protocol (Appendix S1). Further studies were identified by members of the WHO HIV Monitoring Technologies working group and considered for inclusion in the review.

### Study selection

Titles and abstracts were screened for relevance and eligible articles subjected to a full review of the text and assessed against inclusion criteria. The inclusion criteria were: evaluation or comparison of performance of commercially available VL quantification assays or EID assays; evaluation of DBS with valid reference sample using the same VL or EID technology; any HIV-1 subgroup recognition.

### Data extraction

Data were extracted from included studies into an excel file which comprised of general information, study characteristics, participant or sample characteristics, reference and index technology, and outcome data or results including data on accuracy (bias, sensitivity and specificity) and precision (co-efficient of variation). Two independent reviewers extracted data and where discrepancies occurred, agreement was reached through discussion and further article review.

### Quality assessment

Each of the thirteen HIV VL articles and five EID articles included in the review were assessed for quality by two independent reviewers. Twenty four criteria, based on the STARD guidelines, were used to assess the quality of publications [12], [13]. Each reviewer independently scored the publications on the twenty-four quality criteria with a binary yes/no response. In the case of a disagreement, the reviewers discussed the discrepancy until a consensus could be reached.

## Results

### Characteristics of included studies

**Figure 1:**
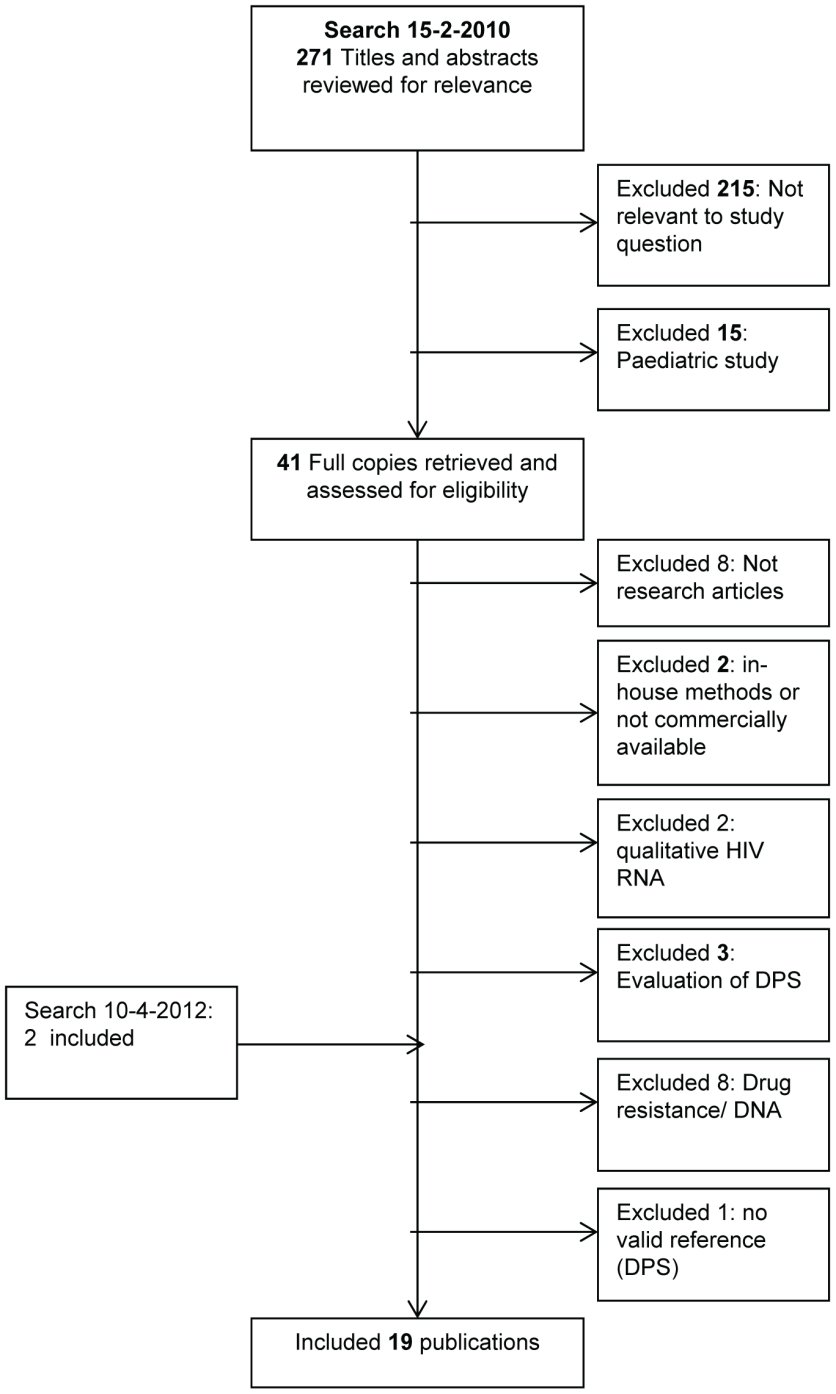
Search algorithm.

For HIV VL, 473 articles were retrieved and 13 articles were included in the final review. For EID, 225 articles were retrieved and 5 articles were included in the final review (Figure 1).

Thirteen studies evaluated the performance of quantitative HIV VL assays, comparing DBS to plasma samples [11], [14]–[25]. All studies used Whatman filter paper 903 (protein saver card, GE healthcare, USA). DBS was compared to plasma samples on Abbott RealTi***m***e HIV-1 assay, M2000 platform (n = 6); four studies used Biomerieux NucliSens easyQ v1.1 (n = 1), and v1.2 (n = 3), Roche COBAS Taqman HIV-1 VL (n = 1), Roche Amplicor Monitor v1.5 (n = 2), and the Versant HIV-1 kPCR (n = 1)(Appendix S2). DBS VL results were compared to plasma results on the same platform. One study conducted plasma and DBS samples in one run, while all remaining evaluations used two runs to quantitate HIV VL using DBS and plasma samples [16]. The sensitivity and specificity of DBS samples on VL assays are summarized in Table 1.

**Table 1:** Sensitivity and Specificity of DBS for HIV VL compared to plasma.

Author	Assay	VL Threshold (copies/mL)	Sample size	Sensitivity (%)*	Specificity (%)*
Lofgren *et al.* [19]	RealTime	1000	176**	100	99
	RealTime	10000	176**	100	100
	RealTime	400	137‡	99	87
	RealTime	5000	137‡	100	97
Pirillo *et al.* [22]	kPCR	37	98	88.2	69.2
	kPCR	5000	98	85.1	96.1
Rottinghaus *et al.* [23]	NucliSens v.1.1	1000	173	77.8	98.1

*sensitivity and specificity given against plasma.

** infants (median 6 months, none on ART).

‡ adults (median 34 years, all on ART).

Five studies evaluated the performance of EID assays, comparing the performance of DBS samples to the reference sample of whole blood or plasma [26]–[29]. Four studies used Whatman 903 filter paper [18], [26], [28], [29] and one study used Whatman No. 1 filter paper [27]. One study evaluated the performance of a HIV VL assay for EID; these results are presented below [19]. Three studies used Amplicor 1.5 assay, 1 used Cobas Taqman and evaluated the Amplicor 1.5 assay, and one used NucliSens EasyQ (Appendix S2). Although Amplicor v1.5 will eventually be phased out by Roche, its performance is still important as it has been the gold standard for the past 20 years and will be the reference assay against which new assays are compared. The samples used for the evaluations were taken from infants younger than 18 months [26], [27], 6 weeks old [28], 8 weeks old [18], and children between 6 weeks and 6 years [29]. The sensitivity and specificity of DBS samples on EID assays are given in Table 2.

**Table 2:** Sensitivity and Specificity of DBS for HIV early infant diagnosis compared to plasma.

Author	Assay	Sample size	Sensitivity (%)*	Specificity (%)*
Anitha *et al.* [26]	Amplicor 1.5	64	100	100
Leelawiwat *et al.* [18]	NucliSens v‡	162	100	100
Nsojo *et al.* [27]	Amplicor 1.5	325	100	99.6
Sherman *et al.* [28]	Amplicor 1.5	2880	100	99.6
Stevens *et al.* [29]	Amplicor 1.5	800	100	99
Stevens *et al.* [29]	COBAS Ampliprep/Taqman	800	100	100

*sensitivity and specificity given against whole blood or plasma.

‡ version number unknown.

### Sample preparation

DBS samples were prepared with either blood collected with EDTA from a venipuncture [11], [14]–[17], [19]–[23], [25], [27]–[29] or heel prick blood [18], [26], [28]. Sample storage conditions for DBS varied from 37°C [17], −4°C [11], −20°C [14], [15], −70°C [18], [27] to ambient temperatures [16], [19]–[22], [24], [25], [28], [29], or any combination of temperatures [23]. Samples in VL studies were humidity controlled with desiccants [14], [15], [19], [22]–[25] but five studies did not specify if desiccants were used [11], [16], [17], [20], [21]. Plasma samples were stored at −70 or −80°C [14], [19], [22], [23], [25], −20°C [11], [15], [16] or not specified [24], [25]. Samples in all EID studies were humidity controlled with desiccants [14], [15], [18], [19], [22]–[29]. Blood samples were stored at −70 or −80°C [18], [27], [29], or not specified [26], [28]. All VL studies included samples with a plasma HIV VL range starting from 2 or 3 log_10_ copies up to 7 log_10_ copies/mL (Appendix S2).

The HIV VLs obtained by DBS samples, which contain approximately 50–100 µl of whole blood, were compared to plasma samples in thirteen studies. Plasma input volumes for the reference VL testing were 100 µl [17], [24], 200 µl [23], 500 µl [14], 600 µl [21], 1000 µl [25] or unspecified [11], [15], [16], [18]–[20], [22]. One study found the mean difference between DBS and plasma VL to be 1.94±0.06 log_10_ c/mL and provided this as a correction factor for DBS VL calculations [20]. An Abbott RealTi***m***e HIV-1 assay protocol for DBS samples is now available, incorporating this correction factor. Haematocrit values can be used in the VL to adjust for the volume of red blood cells, by estimating the amount of plasma in a 50 µl whole blood sample. One study found that haematocrit correction reduced the difference of DBS and plasma VL from −0.43 log_10_ to −0.127 log_10_ [18]. An alternative is to reduce the reference sample volume for a better VL correlation between DBS and plasma [17]. Four out of 13 studies reported that DBS VL were corrected for haematocrit or that DBS were corrected for the smaller sample input volume [14], [18], [20], [23].

### Bias

**Figure 2:**
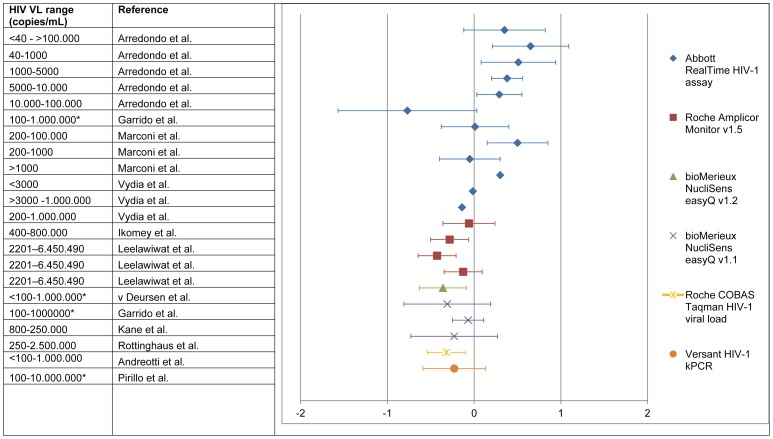
Bias* per HIV viral load range, given for each assay. a haematocrit corrected. b viral load adjusted for smaller input volume. c standard haematocrit value used to correct. * data obtained from Bland-Altman figures. *Bias (DBS-plasma) was plotted as the mean with 1 standard deviation (SD). A negative value represents an HIV VL underestimation compared to plasma while a positive HIV VL bias represents an overestimation of the HIV VL compared to plasma. SD was recalculated to 1 SD when presented as 1.96 or 2SD. In case of bias discrepancy between the text and Bland Altman figure, the bias noted in the text was used (15).

The mean difference, or bias, between DBS and plasma VL measurements (DBS – plasma) ranged from −0.77 (underestimation) to +0.65 log_10_/mL (over estimation) across all studies (Figure 2) 11, 15. For the Abbott RealTi***m***e HIV-1 assay, 51.9% up to 100% of the DBS HIV VL results were within 0.5 log difference of plasma samples [11], [15], [19]–[21], [25]. The percentage of samples within 0.5 log for the Roche COBAS Taqman HIV-1 VL and Versant HIV-1 kPCR were 78.4% [14] and 82.7% [22], respectively. The Biomerieux NucliSens easyQ v1.1 was evaluated by two studies in which 67% [11] and 94% [17] of the results were within 0.5 log. The greatest variance between DBS and plasma was found in studies using the Abbott RealTi***m***e HIV-1 assay (Figure 2).

### Detection limit and sensitivity

The lower detection limit (95% detection rate) of DBS samples with the Abbott RealTi***m***e HIV-1 assay was determined by two different studies to be 550 and 800 c/mL [15], [19]. Andreotti et al. found a low sensitivity when plasma VL samples were below 1,000 c/mL with the Roche COBAS Taqman HIV-1 VL assay (20% detection rate) [14]. Evaluating DBS with the Versant HIV-1 kPCR assay showed that 10 out of 98 samples were not detected when with the corresponding plasma VL was below 597 c/mL [22], which is relatively similar to the lower detection limit found with the Abbott Realti*m*e assay [15], [19].

**Figure 3:**
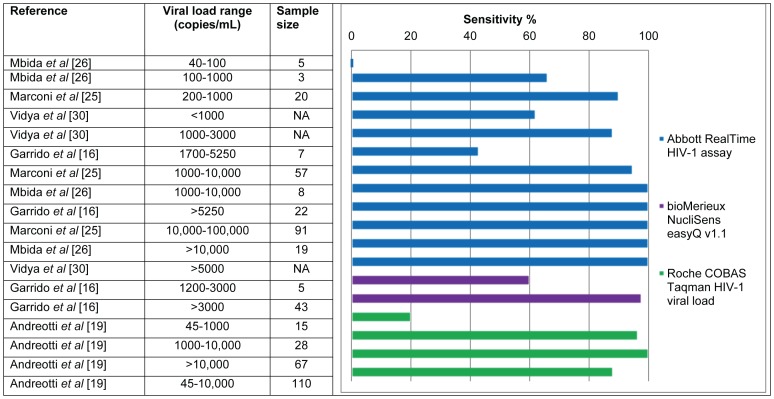
Sensitivity per viral load range of DBS compared to matching plasma samples.

The current clinical threshold proposed by WHO to indicate treatment failure is 5000 c/mL [30]. Depending on the technology and the range of VL in the study population, the sensitivity of DBS VL was found to be 78–100% compared to plasma at VL levels below 1000 copies/ml [23] and varied from 85.1% to 100% in two studies at 5000 c/mL [19], [22]. The specificity of DBS at VL levels of 5000 c/mL was determined in two studies and ranged from 96.1% to 97% [19], [22]. The sensitivities of DBS for samples with a range of plasma VLs are shown in Table 1 and Figure 3.

### Quality assessment

**Figure 4:**
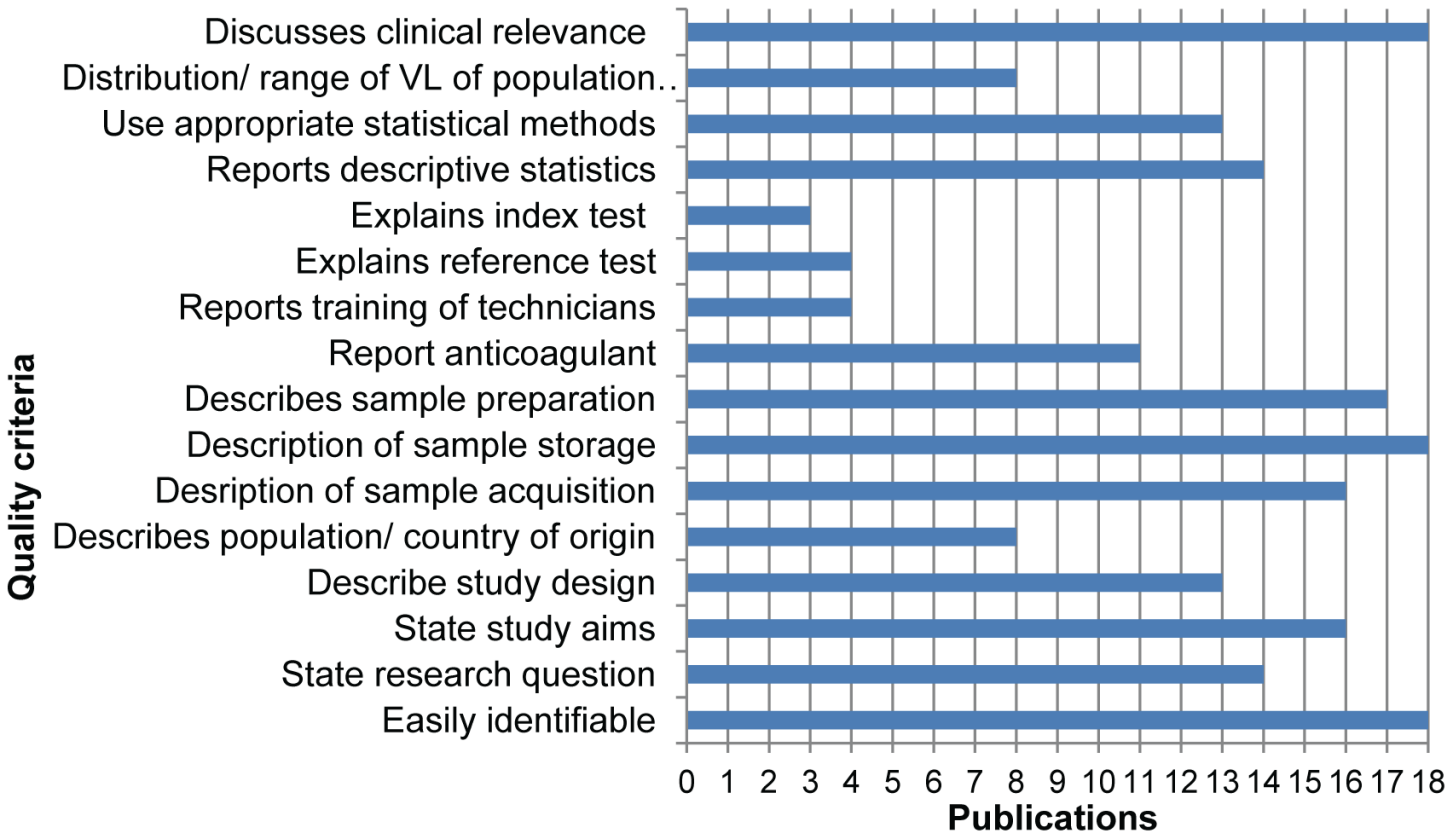
Number of publications matching a selection (18 out of 24) of quality criteria drawn from the STARD guidelines.

All 17 studies described sample storage conditions, discussed the clinical relevance and were easily identified as evaluation studies, with 8 of these describing the population sampled. Furthermore, only eight studies described the distribution or range of HIV VL of the sampled population. Overall, the quality of the papers scored an average of 51% (SD = 9%, range: 25%–63%), based on the STARD guidelines (Figure 4).

## Discussion

DBS samples have been used for many years for the diagnosis of infectious diseases [31]–[33], including the diagnosis of HIV. DBS have been shown to be an extremely useful tool in increasing the access to diagnostic tests, particularly in remote regions. In addition to the practical benefits of using DBS samples, the costs of sample collection and shipment are low compared to plasma samples [34]. Whilst DBS samples appear to be useful for EID and HIV VL measurements, concerns exist with respect to the assays and protocols.

Although literature reviews have been previously published regarding the feasibility of using DBS for HIV VL [4], [35], [36], no literature review has been published that has reviewed HIV EID using DBS samples. The results shown in this study are comparable with other reviews previously published [35], [36]. The review published in 2009 [36] included in-house HIV VL assays, while in this study we focused on commercially available assays and assessed the quality of the papers. Additionally, the previous review included studies evaluating dried serum or plasma spots. Although these alternatives have some advantages over DBS: HIV VL estimations are easier since HIV DNA is not present in plasma but they still require phlebotomy. Serum and plasma spots are more challenging to collect compared to DBS and basic laboratory equipment is required for the separation of blood thus diminishing the value of filter paper.

The majority of studies identified by this review [11], [14]–[24] show that DBS can be used to monitor treatment failure at the current WHO proposed threshold of 5000 c/ml. All assays included in this review are sensitive enough to be used for DBS samples with VL of 3,000 c/mL or greater.

The most important differences in study methods are input volume and the extraction method, both of which contribute to differences in mean bias and variability. Additionally, the method of punching out the filter paper spot, the punch size, elution buffer used, and the sample volume after extraction may alter HIV VL results. Haematocrit correction, quantity of blood and quantity of plasma processed, method of VL calculation, and cell associated HIV RNA and DNA can alter VL measurements. When these critical steps in processing DBS are addressed and resolved, only then will it become possible to reduce result variances and develop uniform protocols to compare sample collection methodologies.

The lower limit of detection in HIV VL quantitation assays when using DBS is 550–1000 c/mL, whereas for plasma it can be as low as 20 c/ml. Even so, the results indicate that DBS VL results are less consistent and vary among the VL platforms. One of the reasons DBS is not, and may never be, as sensitive as plasma in detecting low levels of HIV VL is because of the differences in sample volume between DBS (20–25 µl) and plasma 100 µl to 1.0 mL). When an HIV VL platform has no specific DBS protocol it is necessary to adjust the VL reading for the smaller volume of a DBS sample to obtain a final result comparable to a plasma sample. Unfortunately, most studies did not indicate how they corrected DBS VL results, limiting the ability to further assess this in greater detail.

The DBS VL can be recalculated to plasma VL c/mL by applying the difference between plasma and DBS sample volume. To make the calculation, haematocrit values can be obtained to adjust DBS VL results by calculating the amount of plasma in a DBS sample [18]. Two studies using haematocrit corrections showed that results based on the assumption that 50% of the population is anemic are comparable to corrections based on individual haematocrit values [18], [37]. These calculations are important to compare DBS VL results between authors and to understand the effects of these adjustments on over- or under-quantification of HIV VL results. It is recommended that manufacturers publish DBS protocols to help standardise methodology.

Unlike a plasma VL test which measures only cell free HIV RNA, a DBS VL also measures proviral DNA as well as cell-associated RNA, potentially leading to an overestimation of VL when using DBS [21]. Monleau *et al* treated DBS samples with DNase and found that DNA contributes largely to the HIV VL measurements in DBS samples [38]. The contribution of DNA in HIV VL measurements in DBS has also been noted in samples that are undetectable by plasma VL [22]. The Biomerieux NucliSens EasyQ platform is based on isothermal RNA amplification so HIV DNA is not detected. It can be expected that the levels of proviral DNA and intracellular virions in a sample will differ between patients, and within a single patient due to biological variation. This would imply that a standard correction would be inaccurate and a more detailed procedure would be necessary. Lofgren *et al* [24] showed that when using a cut-off of 400 c/mL, the specificity of DBS is low (87%), as seven samples were over-quantified. As DBS adds more variability to VL results (−0.77 to 0.65 log_10_/mL), it is essential to critically review the laboratory procedures for DBS and minimize variability of each sample process step. Because of the difference in sample volume and the presence of HIV DNA as well as cellular HIV RNA in DBS samples, the quantitation of HIV is complex. In our opinion, a carefully designed and executed reference curve for DBS samples needs to be made for each assay and population.

In contrast to HIV RNA assays, the presence of proviral HIV DNA or cell associated HIV RNA increases the sensitivity of EID assays. DBS samples are regularly used for early infant diagnosis and generally accepted as a sensitive method when venous blood cannot be collected [1], [39]–[41]. Although all studies reviewed here showed good sensitivity, the specificity was slightly less than with fresh blood samples (99–100%). HIV VL in infants is high with inter-quartile ranges of 0.5 million to almost 5 million copies/ml at 6 weeks of age [42] in the absence of prophylactic antiretroviral drugs, suggesting that DBS are a suitable sample [43]. However, as more mothers and infants receive antiretroviral drugs to prevent mother-to-child transmission of HIV, lower HIV RNA levels in infected babies will be observed. Although more evaluations are needed, the use of HIV VL assays for EID with DBS samples could potentially decrease costs, improve access to EID and simplify testing procedures [44], [45].

## Conclusion

There is an urgent need to improve access to VL and EID in resource-limited settings, particularly at peripheral sites where, increasingly, HIV clinical management is being provided. This is consistent with the need to decentralize treatment and care to improve access and retention in care [46]. To date, however, access has been limited by technologies that require sampling at central laboratories.

DBS can be used as an alternative to plasma for HIV VL quantitation based on the suggested threshold of 5,000 c/mL, but challenges surrounding the pre-extraction and analytical stages need to be resolved. Adequate sensitivities can be obtained from 1000 c/mL onwards, and 100% sensitivity is obtained across all platforms when HIV VL is over 3,000 c/mL.

Assays for HIV VL and EID detect nucleic acids and are performed on similar laboratory equipment and HIV VL assays have been evaluated for use for early infant diagnosis. Using the same platform and consumables for two diagnostic tests allows for economies that are particularly advantageous for resource-limited settings. If HIV VL and EID testing were performed on a single technology platform, a standardized approach for sampling, storing, and processing DBS samples would be essential to allow successful implementation.

## Supporting Information

Appendix S1 Search strategy protocol.
(DOCX)

Appendix S2 Summary of studies that evaluated the use of DBS for HIV VL and early infant diagnosis. **+** = Positive. **−** = Negative. NS = Not Stated in article. RT = Room Temperature. RPM = Rotations Per Minute. RBC lysis = red blood cell lysis. ** version of assay is unknown.
(DOC)

Appendix S3 PRISMA Statement.
(DOC)

Checklist S1 PRISMA Checklist.
(DOC)
