# Supplementary material for: Systematic Review of the Use of Dried Blood Spots for Monitoring HIV Viral Load and for Early Infant Diagnosis
Source: PLoS One. 2014 Mar 6;9(3):e86461. doi: 10.1371/journal.pone.0086461 (PMC3945725; doi:10.1371/journal.pone.0086461)
Supplement: Appendix S1 — Search strategy protocol. (DOCX) [file pone.0086461.s001.docx]

**Systematic review of HIV DBS**

**Databases**

EMBASE and MEDLINE were searched

**Search Terms HIV VL DBS**

Search Embase:

("1998/01/01"[Publication Date] : "3000"[Publication Date]) AND ((((dried[tw] OR dry[tw]) AND (spot[tw] OR spots[tw]) AND blood[tw]) OR dbs[tw] OR filter paper[tw] OR filter papers[tw] OR guthrie card*[tw] OR 903 paper[tw]) AND (hiv*[tw] OR human immunodeficiency virus*[tw] OR human immune deficiency virus*[tw] ))

Search medline:

1. ((dried or dry) and blood and spot*)
2. (dbs or "filter paper*" or "guthrie card*" or "903 paper")
3. (hiv* OR "human immunodeficiency virus*" OR "human immune deficiency virus*")
4. 1 OR 2 and 3
5. limit to yr="1998 -Current"

**Search Terms HIV EID**

Search Embase:

("1998/01/01"[Publication Date] : "3000"[Publication Date]) AND ((((dried[tw] OR dry[tw]) AND (spot[tw] OR spots[tw]) AND blood[tw]) OR dbs[tw] OR filter paper[tw] OR filter papers[tw] OR guthrie card*[tw] OR 903 paper[tw]) AND (hiv*[tw] OR human immunodeficiency virus*[tw] OR human immune deficiency virus*[tw] ) AND (DNA* OR RNA OR infant OR neonat*)

Search medline:

1. ((dried or dry) and blood and spot*)
2. (dbs or "filter paper*" or "guthrie card*" or "903 paper")
3. (hiv* OR "human immunodeficiency virus*" OR "human immune deficiency virus*" OR HIV-1)
4. (DNA[tw] OR RNA OR infant OR neonate OR neonatal OR infants*)
5. (“early infant diagnosis” OR EID)
6. limit to yr="1998 -Current"
7. 1 OR 2 AND 3 AND (4 OR 5)

**Date**

1998 to present

**Language**

English

**Publication type/status**

Published works in peer reviewed journals.

**Study selection**

Stage 1: Screening of titles/ abstracts against inclusion criteria.

Titles and abstracts, where available, will be screened and either accepted, rejected as not relevant, or rejected due to failure to meet inclusion criteria (if so, the reason will be specified).

Stage 2: Full papers obtained and assessed against inclusion criteria. Papers will be either accepted or rejected due to failure to meet inclusion criteria and the reason will be specified.

Full papers will be independently assessed by TWO members of the review team and results will be cross-checked and combined.

**Inclusion Criteria**

- Evaluation or comparison of performance of commercially available DNA/RNA assays with DBS and reference sampling methods
- Evaluations based on human clinical or reference materials

**Exclusion criteria**

- Not an evaluation study or not having a correct reference sample or reference method
- In-house developed assays or a no longer commercially available assay
- Studies with other primary aims other than evaluation of DBS for early infant diagnosis with reference samples *
- Studies related to drug resistance screening, genotyping, sequencing, other “non-diagnostics” evaluation studies
- Studies with a population that does not include newborns or infants
- Study written in a language which is not English

**Data Extraction**

| **General Information** |
| --- |
| Date of data extraction |
| Identification features of the study:   - Record number - Author - Article title - Citation |
| **Study Characteristics** |
| Aim/ objectives of the study |
| Filter paper type |
| **Participant/ Sample Characteristics** |
| Characteristics of population from which samples were drawn:   - Age - Sex |
| Number of samples |
| Sample country/ region of origin |
| Sample type (finger prick/ EDTA/..) |
| DBS storage:   - At research site - At laboratory |
| Use of desiccants? |
| Type of reference sample |
| DBS quality check performed? |
| **Technology** |
| Name and manufacturer of assays under evaluation |
| **Extraction method** |
| Extraction volume/adjustments |
| Extraction kit/ method used |
| Detection method |
| If quantitative; adjusted for DBS sample input? |
| **Outcome data/ results** |
| Unit of assessment/ analysis |
| **Outcomes:**  Mean pathogen load + standard deviation  Range of pathogen load  Correlation (r)  Bias (mean difference)  Sensitivity, cut-off  Specificity, cut-off  % CV index test, reference test |
| **Serology:**  Sensitivity, cut-off  Specificity, cut-off  Cut-off adjusted for DBS?  Titre lower limit of detection  Variability, cut-off, replicates |
| For each pre-specified outcome:   - Reported (Y/N) - Definition used in study |
| Additional outcomes reported |
| Details of any additional relevant outcomes reported |

**Quality Assessment**

| **Title/ abstract** | Is the article easily identified as a study of test evaluation? |  |
| --- | --- | --- |
| **Introduction** | Does it clearly state the research question and study aims? |  |
| **Methods** | Do the authors clearly describe the study design? |  |
|  | Are study inclusion and exclusion criteria provided? |  |
|  | Were steps taken to introduce blinding and random allocation where possible and appropriate? |  |
| **Participant/ Sample Characteristics** | Is the population from which the samples were drawn described? |  |
|  | Is the country/ region of sample origin detailed? |  |
|  | Were the samples collected prospectively? |  |
|  | Do the authors describe how the samples were acquired, stored and prepared? |  |
|  | Was the choice of anticoagulant appropriate for the technology? |  |
| **Test methods** | Were staff trained in the use of the technology prior to performing the index and reference tests? |  |
|  | Was the reference standard explained in sufficient detail to be reproduced? |  |
|  | Is the index test explained in sufficient detail to be reproduced? |  |
|  | Did the authors report the number of technicians reading the index/ reference tests? |  |
|  | Was a single sample divided and tested by each technology included in the study or were a different set of samples used for each test? |  |
| Statistical methods | Were the methods used reported in detail? |  |
|  | Were the methods used appropriate? |  |
| **Results** |  |  |
| Participants | Were the demographic characteristics of the population described? |  |
| Test results | Was the distribution/ range of viral load of the sampled population reported? |  |
|  | How were the data presented? |  |
|  | Was subgroup analysis performed for different HIV-1 subtypes? |  |
| **Discussion** | Was the clinical relevance of the study findings discussed? |  |
