# Supplementary material for: Systematic Review of the Use of Dried Blood Spots for Monitoring HIV Viral Load and for Early Infant Diagnosis
Source: PLoS One. 2014 Mar 6;9(3):e86461. doi: 10.1371/journal.pone.0086461 (PMC3945725; doi:10.1371/journal.pone.0086461)
Supplement: Appendix S2 — Summary of studies that evaluated the use of DBS for HIV VL and early infant diagnosis. + = Positive. − = Negative. NS = Not Stated in article. RT = Room Temperature. RPM = Rotations Per Minute. RBC lysis = red blood cell lysis. ** version of assay is unknown. (DOC) [file pone.0086461.s002.doc]

| **Author** | **Sample**  **origin** | **ART +/-** | **# Samples** | **Spot µl** | **# Spots used** | **DBS elution volume** | **Elution process** | **Extraction method** | **Detection Platform**  **(Plasma Input Volume)** |
| --- | --- | --- | --- | --- | --- | --- | --- | --- | --- |
|
| **HIV viral load** | | | | | | | | | |
| Andreotti *et al* | Malawi | 102 -  18 + | 129 | 75 | 1 | 2 ml | Overnight, room T | NucliSens miniMAG | COBAS Taqman**  (500µl) |
| Arredondo *et al* | Spain | 70% - | 154 | 50 | 2 | 2ml | 2 hours, room T | M2000sp | M2000rt  (NS) |
| Garrido *et al (a)* | Spain | NS | 97 | 50 | 1 | 2 ml | 2 hours, gentle rotation | Manual NucliSens kit | NucliSens easyQ v1.1  (NS) |
| Garrido *et al*  *(b)* | Spain | NS | 97 | 50 | 1 | 2 ml | 2 hours, gentle rotation | M2000sp | M2000rt  (NS) |
| Ikomey *et al* | Cameroon | NS | 60 | 50 | NS | 0.9ml RBC lysis | vortex 10 min, centrifuged 10 min | NS | Amplicor Monitor v1.5  (NS) |
| Kane *et al* | Senegal | NS | 41 | 50 | 2 | 2 ml | 30 min, R T | NucliSens MiniMAG | NucliSens easyQ v1.2  (100µl) |
| Leelawiwat *et al* | Thailand | 56+ | 56 | 50 | 1 | 0.9ml | 120 min, 25­^0^C | Manual NucliSens kit | Amplicor Monitor v1.5  (NS) |
| Lofgren *et al* | Tanzania | 73+ | 137 | 50 | 2 | 1.7ml | 2 hours, RT | M2000sp | M2000rt  (600µl) |
| Marconi *et al* | Italy | NS | 168 | 50 | 2 | 2 ml | 2 hours, RT | M2000sp | M2000rt  (1000 µl) |
| Mbida *et al* | Cameroon | 12+ | 41 | 50 | 2 | 1.7 ml | 2 hours | M2000sp | M2000rt  (600µl) |
| Pirillo *et al* | France | NR | 98 | 50 | 1 | 1.25 ml | 30 min, RT | VERSANT preparation module | Versant HIV-1 kPCR  (NS) |
| v Deursen *et al* | The Netherlands | 224+ | 224 | 50 | 2 | 2 ml | 30 min, | NucliSens easy MAG | NucliSens easyQ v2.0  (100µl) |
| Rottinghaus *et al* | Nigeria | 173+ | 173 | 100 | 1 | 2 ml | 30 min, RT | NucliSens easy MAG | NucliSens easyQ v1.1  (200µl) |
| Vidya *et al* | India | NS | 100 | 50 | 2 | 1.75 ml | 2 hours, 56­^0^C | M2000sp | M2000rt  (1000µl) |
| **Early infant diagnosis** | | | | | | | | | |
| Anitha *et al* | India | NR | 64 | NS | 1 | 100 ul | 3 hours, 56­^0^C, 10 min 100­^0^C | Chelex-100 resin | Amplicor 1.5  (NS) |
| Leelawiwat *et al* | Thailand | 56+ | 162 | 50 | 1 | 0.9ml | 2 hours, RT, 80RPM | Manual NucliSens kit | NucliSens NASBA**  (NS) |
| Nsojo *et al* | Tanzania | NS | 325 | 20 | 1 | 200ul | 15 min 60­^0^C, 100­^0^C | NS | Amplicor 1.5  (NS) |
| Sherman *et al* | South Africa | NS | 287 | 20 | 1 | 200ul | 15 min 60­^0^C, 100­^0^C | NS | Amplicor 1.5  (NS) |
| Stevens *et al (a)* | South Africa | NS | 812 | 70 | 7*3.2 mm | 200ul | 15 min 60­^0^C, 100­^0^C | NS | Amplicor 1.5  (NS) |
| Stevens *et al*  *(b)* | South Africa | NS | 812 | 70 | 1 | 1100ul | 10 min, 56­^0^C | Cobas Ampliprep | Taqman**  (NS) |
